# Post-Translational Modifications of the TAK1-TAB Complex

**DOI:** 10.3390/ijms18010205

**Published:** 2017-01-19

**Authors:** Yusuke Hirata, Miki Takahashi, Tohru Morishita, Takuya Noguchi, Atsushi Matsuzawa

**Affiliations:** Laboratory of Health Chemistry, Graduate School of Pharmaceutical Sciences, Tohoku University, 6-3, Aoba, Aramaki, Aoba-ku, Sendai 980-8578, Japan; y-hirata@m.tohoku.ac.jp (Y.H.); miki.t@dc.tohoku.ac.jp (M.T.); tmrst.1119@gmail.com (T.M.)

**Keywords:** TAK1, TAB1, TAB2, TAB3, post-translational modification, NF-κB, MAP kinase

## Abstract

Transforming growth factor-β (TGF-β)-activated kinase 1 (TAK1) is a member of the mitogen-activated protein kinase kinase kinase (MAPKKK) family that is activated by growth factors and cytokines such as TGF-β, IL-1β, and TNF-α, and mediates a wide range of biological processes through activation of the nuclear factor-κB (NF-κB) and the mitogen-activated protein (MAP) kinase signaling pathways. It is well established that activation status of TAK1 is tightly regulated by forming a complex with its binding partners, TAK1-binding proteins (TAB1, TAB2, and TAB3). Interestingly, recent evidence indicates the importance of post-translational modifications (PTMs) of TAK1 and TABs in the regulation of TAK1 activation. To date, a number of PTMs of TAK1 and TABs have been revealed, and these PTMs appear to fine-tune and coordinate TAK1 activities depending on the cellular context. This review therefore focuses on recent advances in the understanding of the PTMs of the TAK1-TAB complex.

## 1. Introduction

**Figure 1:**
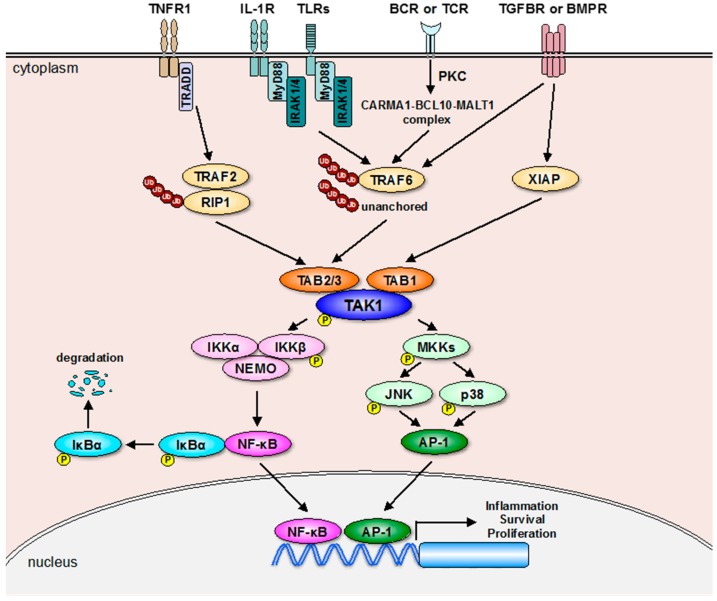
Receptor-mediated TAK1 signaling pathways. Receptor-mediated activation of TAK1 is mainly mediated by the E3 ubiquitin ligase TRAF2 or TRAF6 that promotes formation of the TAK1-TAB complex. On the other hand, the E3 ubiquitin ligase XIAP activates TAK1 through the direct interaction with TAB1 downstream of TGFBR or BMPR activation probably without E3 ubiquitin ligase activity. Moreover, a recent report showed that unanchored K63-linked polyubiquitin chains are sufficient to activate TAK1 [7]. TAK1 activated by these multiple mechanisms upregulates NF-κB- and AP-1-depenedent gene expression through activating the NF-κB and MAP kinase (JNK and p38) pathways. The arrows show positive regulation and “p” indicates phosphorylation.

Transforming growth factor-β (TGF-β)-activated kinase 1 (TAK1, also known as MAP3K7), a member of the mitogen-activated protein (MAP) kinase kinase kinase (MAP3K) family, was originally identified as a protein kinase that is activated by TGF-β and bone morphogenic protein (BMP) [1]. Subsequent studies have revealed that TAK1 is activated by a wide variety of proinflammatory mediators, such as tumor necrosis factor (TNF)-α, interleukin (IL)-1β, toll-like receptor (TLR) ligands, and T-cell receptor (TCR) and B-cell receptor (BCR) antigens [2,3]. In these, inflammatory signaling, nuclear factor-κB (NF-κB), and transcription factor activator protein-1 (AP-1) are major transcriptional factors to induce diverse biological responses including cell proliferation, differentiation, survival, and innate and acquired immunity [4]. Importantly, the activation of these two transcriptional factors is predominantly regulated by TAK1-dependent signaling cascade [2,3] (Figure 1). Therefore, TAK1 is perceived as an essential component in inflammatory signaling. On the other hand, it has been reported that TAK1 is activated by various stresses including DNA damage and osmotic shock, indicating the involvement of TAK1 in stress-response signaling [2,5,6]. Thus, accumulating evidence has indicted the importance of TAK1 as a multifunctional kinase that can respond to a wide range of stimuli (Table 1).

**Figure 2:**
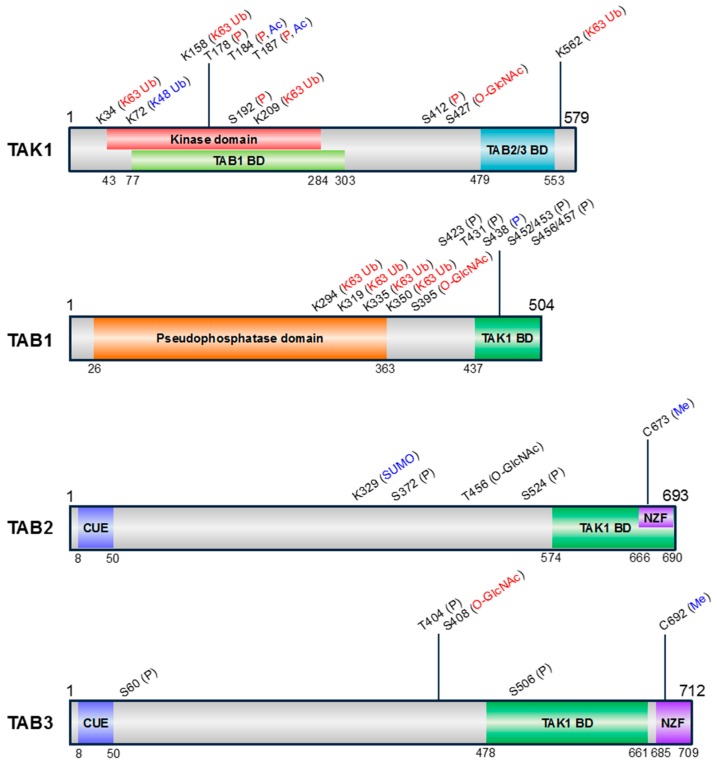
Schematic representation of the domain structures and PTM sites of human TAK1 and TABs. The amino acid positions of each domain are indicated below the structures. Information of kinase domain of TAK1, pseudophosphatase domain of TAB1, coupling of ubiquitin conjugation to endoplasmic reticulum degradation (CUE), and Npl4 zinc finger (NZF) ubiquitin binding domains of TAB2 and TAB3 was obtained by SMART (simple modular architecture research tool) (http://smart.embl-heidelberg.de/). Information of TABs binding domain (BD) in TAK1 [14,18] and TAK1 BD in TABs [9,14,19] was obtained from previous studies analyzing the individual domains. The pseudophosphatase domain of TAB1 has a similar structure to protein phosphatase 2C (PP2C), despite lacking phosphatase activity [20]. The types of PTMs are indicated above the corresponding positions and are color-coded according to the effect on TAK1 activity (positive, red; negative, blue; unknown, black). P, phosphorylation; Ub, ubiquitination; SUMO, SUMOylation; Ac, acetylation; *O*-GlcNAc, *O*-GlcNAcylation; Me, Methylation.

TAK1 interacts with TAK1-binding proteins TAB1, TAB2, and TAB3. The formation of the TAK1-TAB1-TAB2 or TAK1-TAB1-TAB3 complex is required for the autophosphorylation-induced activation of TAK1 [8,9,10,11,12]. TAK1 binds to TABs through the TAK1 binding domain (TAK1 BD) located at the C-terminus of TABs [8,9,10,11,12]. Each TAK1 BD contains α-helices that are necessary for the interaction with TAK1, despite the low sequence homology [9,12,13,14]. On the one hand, TAB1 constitutively binds to the N-terminus of TAK1, whereas TAB2 and TAB3 bind to the C-terminus of TAK1 in a context-dependent manner (Figure 2). In addition, TAK1 deletion mutant lacking N-terminal 22 amino acids exhibits constitutive activity [1], and a peptide containing N-terminal 77 amino acids of TAK1 can inhibit TAK1 activation [15]. Therefore, the N-terminal region of TAK1 has been suggested to be an autoinhibitory domain. A chimeric protein containing TAK1 kinase domain and TAK1 BD of TAB1 has been reported to be constitutively active [16], and the crystal structure of which has been reported [17]. However, the crystal structure of the full-length TAK1-TABs complex has yet to be determined, and further structural insight into TAK1 activation involving the TAK1-TABs complex has not been provided.

Although the upstream regulators of TAK1 depend on the type of stimulus, the interaction with TABs is an essential step for TAK1 activation. Interestingly, emerging studies have demonstrated the existence of post-translational modifications (PTMs) of TAK1 and TABs, indicating that PTMs are functionally involved in the processes of TAK1 activation including the formation of the TAK1-TAB complex. In this review, we thus focus on the PTMs of the TAK1-TAB complex, which may play critical roles in the regulation of TAK1-dependent signaling.

## 2. TAK1 Signaling Pathway

As shown in Figure 1, receptor-mediated TAK1 activation mostly depends on the E3 ubiquitin ligase activity of TNF-receptor-associated factor (TRAF) 2 or 6 [3,21,22]. Once TNF-α binds to TNF receptor 1 (TNFR1), the adaptor molecule TNFR1-associated DEATH domain protein (TRADD) is recruited to TNFR1, which in turn binds to TRAF2 and receptor-interacting protein 1 (RIP1), and promotes TRAF2-mediated K63-linked polyubiquitination of RIP1 [3,22]. After the K63-linked polyubiquitination of RIP1, the polyubiquitin chains of RIP1 bind to the C-terminal Npl4 zinc finger (NZF) domain of TAB2 and TAB3, which allows the autophosphorylation-dependent activation of TAK1 to occurr after conformational changes [21,23,24]. The structural basis for the interaction between K63-linked polyubiquitin chains and the NZF domains of TAB2 and TAB3 has been determined by crystal structure analysis [25,26]. The K63-linked polyubiquitination of RIP1 also functions as a scaffold where TAK1 phosphorylates its substrates such as the inhibitor of nuclear factor κB kinase β (IKKβ) MAP kinase kinases (MKKs) including MKK3, MKK4, MKK6, and MKK7. IKKβ is a component of the IKK complex consisting of IKKα, IKKβ, and IKKγ (also referred to as NF-κB essential modulator (NEMO)) that is essential for the activation of NF-κB [4]. MKKs activated by TAK1 upregulate the transcriptional activity of AP-1 via activation of the p38 and JNK pathways [27]. Thus, the K63-linked polyubiquitination mediated by TRAF2 plays a critical role in TNFR1-induced TAK1 activation.

As well as TRAF2, TRAF6 mediates the receptor signaling pathways that are, in particular, activated by IL-1β, TLR ligands, TGF-β, BCR/TCR antigens, and BMP, and it has been well characterized that the ligand-stimulated receptors activate TRAF6 by distinct mechanisms [2,3,28]. For TRAF6 activation, IL-1 receptor (IL-1R), and TLR request myeloid differentiation primary response gene 88 (*MyD88*), interleukin-1-receptor-associated kinase 1 (IRAK1), and IRAK4, whereas BCR and TCR request formation of the signaling complex, including caspase recruitment domain family member 11 (CARD11, also called CARMA1), B-cell CLL/lymphoma 10 (BCL10), and mucosaassociated lymphoid tissue lymphoma translocation gene 1 (MALT1), following the activation of the phospholipase C (PLC) and protein kinase C (PKC) [3,21,29,30]. TRAF6 recruited to the receptor complex undergoes autoubiquitination, and the C-terminal NZF domain of TAB2 and TAB3 binds to the polyubiquitin chains of TRAF6, resulting in the activation of TAK1. On the other hand, TGF-β receptor (TGFBR) appears to directly interact with and induce autoubiquitination of TRAF6, although the involvement of TRAF6 in BMP receptor (BMPR)-induced TAK1 activation is unclear [31,32]. Alternatively, an E3 ubiquitin ligase X-linked inhibitor of apoptosis protein (XIAP) participates in the TAK1 activation downstream of TGFBR or BMPR through the formation of the XIAP-TAB1-TAK1 complex, likely in a ubiquitination-independent mechanism [33,34].

It has been shown that DNA damage-induced TAK1 activation is also mediated by K63-linked polyubiquitination. In response to genotoxic drugs such as etoposide and doxorubicin, ataxia-telangiectasia-mutated (ATM) kinase is exported from the nucleus to the cytosol, triggering the formation of the ATM-NEMO-XIAP-Ubc13 complex [35]. The complex formation increases the E3 ubiquitin ligase activity of XIAP, which induces the K63-linked polyubiquitination of scaffold protein, glutamate, leucine, lysine, and serine-rich protein (ELKS), and then the K63-linked polyubiquitin chains of ELKS provide opportunities for the TAK1, TAB2, and TAB3 interaction and subsequent TAK1 activation [35]. Etoposide and doxorubicin also induce the recruitment of RIP1 to the ATM-NEMO complex and then promote the K63-linked polyubiquitination of RIP1 [36]. The RIP1 ubiquitination might be required for TAK1 activation, since both etoposide and doxorubicin failed to activate TAK1 in the absence of RIP1 [36]. Moreover, K63-linked polyubiquitination and monoubiquitination of NEMO mediated by TRAF6 and cellular inhibitor of apoptosis (cIAP), respectively, are required for TAK1 activation induced by ionizing radiation [6]. Thus, accumulating evidence indicates that the multiple molecular mechanisms associated with the E3 ubiquitin ligases are involved in DNA damage-induced TAK1 activation.

It has been reported that osmotic stress, hypoxia, and Wingless and int-1 (Wnt) signaling also activate TAK1, although the mechanisms are not well defined [2]. In the noncanonical Wnt signaling pathway, Ca^2+^/calmodulin-dependent protein kinase II (CaMKII) promotes TAK1-mediated activation of nemo-like kinase (NLK), which functions as a negative feedback regulator of Wnt signaling by suppressing transcriptional activity of β-catenin [37,38]. However, the molecular mechanisms by which CaMKII activates TAK1 remain unclear, although several studies have supported the importance of the TAK1-NLK axis in the regulation of Wnt signaling [39,40,41]. Hypoxia also induces TAK1 activation via CaMKII, which contributes to hypoxia-induced NF-κB activation [42]. The hypoxia-induced activation of TAK1 requires XIAP, Ubc13, c-Jun-amino-terminal kinase-interacting protein (JIP1), and vaccinia-related kinase 1 (VRK1), while the requirement of TABs for the TAK1 activation remains contentious [43,44]. Osmotic stress seems to activate the JNK pathway via TAK1, because JNK activation was significantly suppressed by TAK1 knockout in keratinocytes and mouse embryo fibroblasts (MEFs) [45]. Moreover, several lines of evidence have shown that TAK1 plays a role as an upstream kinase of AMP-activated protein kinase (AMPK) in response to oxidative stress [46], TNF-α-related apoptosis-inducing ligand (TRAIL) [47], TNF-α, and lipopolysaccharide (LPS) [48]. However, the activation mechanisms of TAK1 induced by these stimuli remain elusive.

## 3. PTMs of TAK1 and TABs

To date, a wide variety of PTMs of TAK1 and TABs have been reported, which may play a critical role in modulating the TAK1-TAB complex formation and TAK1 activation has been reported (Figure 2 and Table 2). We summarize the current understanding of these PTMs of TAK1 and TABs. Question marks indicate that it is unknown.

### 3.1. Phosphorylation

Once activated, TAK1 autophosphorylates four serine (Ser) or threonine (Thr) residues, Thr178, Thr184, Thr187, and Ser192 within the activation loop, and the activation status of TAK1 is frequently monitored by phospho-specific antibody against Thr187 of TAK1 [19,57,66,92,93]. A mutational analysis revealed that TAK1 first autophosphorylates Ser192 in vitro in the presence of TAB1, followed by sequential phosphorylation of Thr178, Thr187, and finally Thr184, although it was not investigated in physiological conditions [92]. A recent study has demonstrated a mechanism of Thr187 phosphorylation mediated by tumor progression locus 2 (TPL2, also known as cancer osaka thyroid (COT) or MAP3K8) in response to IL-17 [59]. IL-17-induced Thr187 phosphorylation of TAK1 was diminished in TPL2 knockout cells, and an in vitro kinase assay showed the ability of TPL2 to phosphorylate TAK1 at Thr187, indicating that TPL2 directly phosphorylates TAK1 in response to IL-17 stimulation, and thereby promotes the activation of downstream signaling [59]. Systemic knockout of TPL2 or astrocyte-specific knockout of TAK1 decreased severity of experimental autoimmune encephalomyelitis (EAE), suggesting that the TPL2-TAK1 axis contributes to the pathogenesis of EAE [59]. On the other hand, protein kinase A (PKA)-mediated phosphorylation of TAK1 at Ser412 positively regulates TAK1 activation [69]. A mutant form of TAK1 in which Ser412 was substituted by alanine (TAK1 S412A) exhibits dominant-negative effects that inhibit TAK1-mediated osteoclast differentiation and cytokine production in RAW264.7 cells, indicating that the phosphorylation of Ser412 is required for TAK1 activation [69]. Moreover, ectopic expression of TAK1 S412A mutant suppressed the activation of the p38, JNK, and NF-κB pathways, supporting the requirement of Ser412 phosphorylation for TAK1 activation [70].

It has been reported that the phosphorylation of Thr187 and Ser412 is negatively regulated by the phosphatases. TNF-α and IL-1β induce the interaction of TAK1 with dual-specificity phosphatase 14 (DUSP14, also known as MKP6), promoting the dephosphorylation of TAK1 at Thr187 [60]. The dephosphorylation of TAK1 at Thr187 is also mediated by PP2A in TGF-β signaling, and PP2Cβ-1, PP2Cε and PP6 in IL-1β signaling [61,62,63,64]. Moreover, hypertrophic stimuli induce the dephosphorylation of TAK1 at Thr187 via a mechanism involving Ca^2+^-dependent phosphatase calcineurin (CaN) and regulator of calcineurin (RCAN) [65]. TLR-dependent TAK1 phosphorylation of Ser412 is dephosphorylated by PP1 cooperatively with growth arrest and DNA damage-inducible protein 34 (GADD34), leading to termination of TAK1-dependent inflammatory responses [71].

TABs are also regulated by phosphorylation-dependent signaling. In case of TAB1, seven residues (Ser423, Thr431, Ser438, Ser452, Ser453, Ser456, and Ser457) are phosphorylated, although the functional roles of these phosphorylations for TAK1 activation are not established except for Ser438 (Table 1). TAK1 activation induced by TNF-α, IL-1α, or osmotic stress was enhanced in p38α knockout MEFs, suggesting that p38α negatively regulates TAK1 activity [5]. Because p38 inhibitor SB203580 can strongly suppress the phosphorylation of TAB1 at Ser423 and Thr431, the phosphorylations are most likely to be mediated by p38 [5]. These findings raise the possibility that p38 inactivates TAK1 through the phosphorylation of TAB1 at Ser423 and Thr431. Moreover, it has been reported that four serine residues of TAB1 (Ser452, Ser453, Ser456, and Ser457) are phosphorylated by p38 or TAK1 in response to IL-1α, anisomycin, and sorbitol [83]. The phosphorylation of TAB1 at Ser438 induced by IL-1α and osmotic stress was suppressed by PD184352 that is an inhibitor of the extracellular signal-regulated kinase (ERK) pathway, or JNK1 and JNK2 double knockout, suggesting involvement of these pathways in the TAB1 phosphorylation [5,81]. On the contrary, a phosphatase DUSP14 dephosphorylates Ser438 of TAB1, and thereby negatively regulates TCR signaling [82]. Indeed, DUSP14 knockout increased phosphorylation levels of TAB1 Ser438, and enhanced in vivo immune responses and susceptibility to EAE [82].

TAB2 and TAB3 are phosphorylated at Ser372 and Ser524, and Ser60, Thr404, and Ser506, respectively, in response to IL-1α or IL-1β [64]. However, as well as the TAB1 phosphorylation, the functional roles of these phosphorylations remain to be determined. Nonetheless, the phosphorylation of TAB3 at Ser60, Thr404, and Ser506 was abrogated by p38 inhibitors, whereas the phosphorylation of TAB2 at Ser372 and Ser524 was not affected by the inhibition of the ERK, p38, or JNK activation [81]. On the other hand, multiple phosphorylation sites of TAK1 and TAB1 were identified by using mass spectrometry, and whose phosphorylations are induced by co-expression of type 2A phosphatase-interacting protein (TIP) that directly interacts and promotes TAK1 activation, like the TABs [94]. Taken together, the activation status of TAK1 seems to be regulated by a delicate balance of phosphorylation and dephosphorylation of TAK1 itself and TABs, yet further studies are necessary for a full understanding of the phosphorylation-dependent modulation of TAK1 activation.

### 3.2. Ubiquitination

In addition to phosphorylation, ubiquitination is one of the most extensively studied PTMs [95,96]. Ubiquitination is a dynamic process that is mediated by the ubiquitin-activating enzyme (E1), ubiquitin-conjugating enzyme (E2), and ubiquitin ligase (E3), and readily reversed by a family of deubiquitinating enzymes (DUBs). According to the linkage type, ubiquitination regulates distinct biological processes. In particular, lysine 48 (K48)-linked polyubiquitin chains serve as a marker for proteasomal degradation, whereas lysine 63 (K63)-linked polyubiquitin chains regulate diverse cellular functions [97,98]. To date, four lysine (Lys) residues—Lys34, Lys158, Lys209, and Lys562—of TAK1, have been identified as potential sites of K63-linked polyubiquitination [31,49,52,53,54,55,56,67,68,73]. In response to TNF-α, IL-1β, and TGF-β, TRAF2 or TRAF6 mediates K63-linked polyubiquitination of TAK1 at Lys158 [53,54]. A really interesting new gene (RING) finger protein tripartite motif 8 (TRIM8) is also involved in K63-linked polyubiquitination at Lys158 upon TNFR1 or IL-1R activation [55]. Moreover, infection of *Helicobacter pylori* promotes K63-linked polyubiquitination of TAK1 at Lys158 through the virulence factor cytotoxin-associated gene A (CagA) [56]. These reports suggest that the K63-linked polyubiquitination of Lys158 positively regulates TAK1-mediated signaling [52,53,54,55,56]. On the other hand, polyubiquitination of TAK1 at Lys34 and Lys209 remains controversial. It has been demonstrated that TRAF6-mediated polyubiquitination of TAK1 at Lys34 promotes p38 and NF-κB activation, which was impaired by substitution of Lys34 for arginine (K34R) when HEK293 cells and MEFs were treated with LPS, TNF-α, TGF-β, or IL-1β [31,49]. TRAF6 also promotes Lys209 polyubiquitination of TAK1 upon IL-1R activation, leading to formation of the TRAF6-TAK1-MAP kinase kinase kinase 3 (MEKK3) complex that contributes to the sustained activation of NF-κB following TAK1 activation in HEK293T cells and MEFs [67]. Furthermore, Sef-S, a short splice isoform of similar expression to *fgf* genes (Sef), activates TAK1 through promoting K63-linked polyubiquitination of Lys209 [68]. However, several lines of evidence demonstrate that TAK1 K34R and K209R mutants were activated to the same extent as TAK1 wild-type (WT), showing that the polyubiquitination of TAK1 at Lys34 and Lys209 is not necessary for TAK1 activation [52,54]. Thus, further studies are needed to account for the discrepancy in the functions of K63-linked polyubiquitination at Lys34 and Lys209. Meanwhile, Lys562 has emerged as a novel K63-linked polyubiquitination site of TAK1 induced by LPS [73]. TAK1 K562R mutant showed diminished activation of TAK1 and MAP kinases, suggesting that the polyubiquitination of TAK1 at Lys562 positively regulates TAK1 activation [73].

USP4 and USP18 have been identified as DUBs that downregulate TAK1-mediatied signaling through the deubiquitination of TAK1 [74,75,76]. A subsequent study revealed that USP4 cleaves K63-linked polyubiquitin chains of TAK1 at K158, whereas targets of USP18 still remain elusive [50]. Cylindromatosis (CYLD) was identified as a key player in preventing spontaneous activation of TAK1 through promoting deubiquitination of TAK1 downstream of TCR signaling [77]. Moreover, a recent report has shown a sequential mechanism for TAK1 inactivation mediated by the CYLD-itchy E3 ubiquitin protein ligase (ITCH) complex [78]. CYLD first cleaves K63-linked polyubiquitin chains, and then ITCH secondary catalyzes K48-linked polyubiquitination of TAK1, resulting in the downregulation of both TAK1 activation and expression [78]. In addition, later reports have revealed that Lys72 of TAK1 is responsible for the K48-linked polyubiquitination, which is mediated by ITCH at least in the presence of doxorubicin [50,51]. Deficiency in ITCH or CYLD causes sustained activation of TAK1 and increased cytokine production in bone marrow-derived macrophages, and tumorigenesis and metastasis of transplanted Lewis lung carcinoma, suggesting that sustained activation of TAK1 leads to the progression of non-small-cell lung cancer (NSCLC) [78].

K63-linked polyubiquitination of TAB1 is mediated by plant homeodomain (PHD) of mitogen-activated protein kinase kinase kinase 1 (MEKK1) possessing E3 ubiquitin ligase activity [79]. TGF-β induces MEKK1 PHD-mediated TAB1 polyubiquitination at four lysine residues (Lys294, Lys319, Lys335, and Lys350), and a non-ubiquitinated form of TAB1 mutant fails to activate TAK1 and MAP kinases in response to TGF-β [79]. As is the case for TAK1, ITCH can catalyze K48-linked polyubiquitination of TAB1, whereas the site of ubiquitination is not determined [84]. Ring-B-box-coiled-coil (RBCC) protein interacting with protein kinase C1 (RBCK1), possessing E3 ubiquitin ligase activity, was identified as an interacting protein of TAB2 and TAB3 [91]. Ectopic expression of RBCK1 promoted ubiquitination and proteasome-dependent degradation of TAB2 and TAB3, and knockdown of RBCK1 increased NF-κB activation induced by IL-1β and TNF-α, suggesting that RBCK1 negatively regulates TAK1 signaling via K48-linked polyubiquitination and degradation of TAB2 and TAB3 [91]. However, K48-linked polyubiquitination sites of TAB2 and TAB3 remain unidentified.

Several lines of evidence have indicated the involvement of lysosomal degradation in regulating expression of TAK1 and TABs. TRIM30α induced by TLR activation interacts with and promotes degradation of TAB2 and TAB3, which most likely functions as a negative feedback mechanism for TLR signaling [99]. Interestingly, TRIM30α-mediated degradation of TAB2 and TAB3 was blocked by lysosome inhibitors but not proteasome inhibitors, suggesting that TRIM30α mediates lysosomal degradation of TAB2 and TAB3 [99]. In addition, other RING finger proteins, such as TRIM38, TRIM22, and RING finger protein 4 (RNF4), have been identified to contribute to the lysosomal degradation of TAB2 or TAB3 [86,100,101]. Since protein ubiquitination serves as a signal for not only proteosomal degradation but also selective autophagic degradation, the ubiquitin-dependent regulation of the TAK1-TAB complex might contribute to the process of selective autophagy [87,102]. Future studies will reveal the mechanisms by which selective autophagy regulates expression levels of the TAK1-TAB complex.

### 3.3. SUMOylation

The small ubiquitin-like modifier (SUMO) is a ubiquitin-like protein that covalently conjugates a number of target proteins. As well as ubiquitination, SUMOylation is also a dynamic process that is mediated by activating (E1), conjugating (E2), and ligating (E3) enzymes, and regulates a wide range of cellular functions [103]. Among the components of the TAK1-TAB complex, only TAB2 has been reported to be SUMOylated [85]. Lys329 of TAB2 was identified to be SUMOylated by a SUMO E3 ligase protein inhibitor of activated STAT 3 (PIAS3), and the mutation of Lys329 enhanced AP-1 activation, suggesting that the SUMOylation of TAB2 at Lys329 negatively regulates TAK1 activation [85].

### 3.4. Acetylation

Amino-terminal acetylation is a most ubiquitous protein modification in eukaryotes. In humans, 80%–90% of proteins are co-translationally acetylated at the α-amino acid group of the N-terminal amino acid [104]. Furthermore, a number of proteins are post-translationally acetylated at the ε-amino acid group of Lys residues, which regulates diverse protein functions and cellular processes [104]. Notably, a bacterial effector from *Yersinia Pestis* (YopJ) has been identified as an acetyltransferase that mediates *O*-acetylation at the phosphorylation sites of several kinases, including MKK6, IKKα, and IKKβ, thus preventing phosphorylation and activation of these kinases [105,106]. A recent study using *Drosophila* revealed that YopJ negatively regulates NF-κB signaling by acetylation of *Drosophila* TAK1 targeting the serine and threonine residues essential for its activation [58]. It was further demonstrated that YopJ acetylates human TAK1 at Thr184 and Thr187, whose phosphorylation is essential for TAK1 activation as mentioned above [58]. Ectopic expression of WT YopJ, but not catalytically inactive mutant YopJ, abrogates Thr187 phosphorylation of TAK1 in HEK293T cells, suggesting that YopJ-mediated acetylation directly counteracts TAK1 activation [58].

### 3.5. Methylation

Protein methylation is a PTM through which methyl groups are transferred from *S*-adenosyl-l-methionine to nitrogen side-chains in arginine or lysine residues by methyltransferases [107]. Protein methylation was first identified as a modification that regulates chromatin remodeling and gene transcription, and is currently perceived as a PTM that can potentially regulate signal transduction pathways in a similar manner as protein phosphorylation [107]. A recent work has demonstrated that cysteine (Cys) methylation of TAB2 and TAB3 is mediated by bacterial type-III-secreted effector NleE from enteropathogenic *Escherichia coli* [89]. NleE harbors methyltransferase activity that specifically modifies zinc-coordinating Cys673 of TAB2 and Cys692 of TAB3 in the NZF domains [89]. NleE-mediated Cys methylation disrupts the interaction of TAB2 and TAB3 with K63-linked polyubiquitin chains, leading to inactivation of TAK1-mediated inflammatory responses [89]. Moreover, a subsequent study revealed the critical motif ^49^GITR^52^ in NleE for binding to TAB2 and TAB3, and NleE mutants lacking ^49^GITR^52^ failed to methylate TAB3 [90]. Similar results were observed for the NleE homolog, OspZ, from *Shigella flexneri* 6 that also bounds TAB3 through the ^49^GITR^52^ motif, suggesting that the substrate-binding motif in NleE and OspZ is critical for the downregulation of TAK1-mediated signaling [90].

### 3.6. O-GlcNAcylation

*O*-GlcNAcylation is the process where *O*-Linked β-*N*-acetylglucosamine (*O*-GlcNAc) is transferred from uridine diphosphate *N*-acetylglucosamine (UDP-GlcNAc) to serine or threonine residue in proteins by *O*-GlcNAc transferase (OGT) [108]. *O*-GlcNAcylation has recently emerged as a new type of PTM that positively regulates TAK1 activation. TAK1 Ser427 is modified with *O*-GlcNAc in response to IL-1α and osmotic stress, and the Ser-substituted mutant reduces TAK1 autophosphorylation and following activation of the downstream signaling without affecting the interaction between TAK1 and TAB1 [72]. IL-1α and osmotic stress also induce *O*-GlcNAcylation of TAB1 at Ser395 [80]. TAB1 S395A mutant inhibits TAK1 autophosphorylation, and subsequent activation of the JNK and NF-κB pathways [80]. Alternatively, it has been demonstrated that *O*-GlcNAcylation of TAB3 at Ser408 was promoted in triple-negative breast cancer (TNBC) cells, and *O*-GlcNAcylation levels of TAB3 at Ser408 correlated well with the prognosis of TNBC patients [88]. Moreover, stable transfection of TAB3 S408A mutant suppressed IL-1β-induced activation of TAK1, which leads to decreased cell migration and invasion both in vitro and in vivo [88]. These findings suggest that the *O*-GlcNAcylation of TAB3 at Ser408 is required for TAK1 activation, and the hyperactivation of TAK1 induced by the excessive *O*-GlcNAcylation of TAB3 is involved in tumor progression [88]. Finally, although the functional importance is not clarified yet, other *O*-GlcNAcylation sites of TABs have been identified by mass spectrometry analysis [109,110,111].

## 4. Conclusions

It has been demonstrated by a series of knockout studies that the TAK1-TAB complex functions as a critical signaling hub, which mediates a wide range of biological processes, and which has been demonstrated by a series of knockout studies. The knockout of either TAK1, TAB1, or TAB2 in mice leads to embryonic lethality due to developmental defects in mice [112,113,114,115]. Instead, analyses of conditional knockout mice lacking TAK1 have revealed that TAK1 deficiency causes various types of defects—such as abnormal cell differentiation, increased cell death, and decreased inflammatory responses—due to impaired TAK1 signaling [116]. On the other hand, TAK1 signaling is also positively associated with a variety of disorders; TAK1 deficiency or inhibition suppresses renal inflammation and fibrosis [117], contact hypersensitivity response [118], and neuronal death in cerebral ischemia [119]. Moreover, excessive activation of TAK1 links to the pathogenesis of autoimmune diseases, and cancer development and progression [116]. Knockout of DUSP14, and that of ITCH or CYLD promotes progression of EAE and lung cancer, respectively, accompanied with sustained activation of TAK1 signaling [78,82]. The level of *O*-GlcNAcylation of TAB3 at Ser408, positively regulating TAK1 activation, correlated well with the prognosis of TNBC patients [88]. Importantly, a recent genomic analysis has shown that mutations in *MAP3K7* (encoding TAK1) or *TAB2* found in Frontometaphyseal dysplasia (FMD)—one of the otopalatodigital syndrome spectrum disorders—cause increased TAK1 autophosphorylation at Thr187 and activation of MAP kinase and NF-κB pathway, suggesting that excessive activation of TAK1 accounts for a human developmental disorder [120].

Thus, accumulating evidence has demonstrated the physiological and pathological significance of TAK1 signaling, and suggested that dysregulation of the PTMs of the TAK1-TAB complex contributes to the pathogenesis of TAK1-related disorders [116]. Therefore, comprehensive characterization of the PTMs of the TAK1-TAB complex will lead to the development of a new therapeutic strategy to overcome TAK1-related diseases.

## Figures and Tables

**Table 1:** Major stimuli inducing TAK1 activation.

Receptor-Mediated Signaling	Stress Response
IL-1α, IL-1β	DNA damage
TLR ligands	Oxidative stress
TNF-α	Osmotic stress
TGF-β	Hypoxia
BMP	–
T-cell antigens	–
B-cell antigens	–
Wnt	–

**Table 2:** PTMs of TAK1 and TABs.

Protein	Site	PTM	Reaction	Catalyzed By	Effect on TAK1 Activity	Inducing Stimuli	Reference
TAK1	K34	K63 Ub	ubiquitination	TRAF6	+	TGF-β, TNF-α, LPS, IL-1β	[31,49]
	K72	K48 Ub	ubiquitination	ITCH	–	TNF-α, Doxorubicin	[50,51]
	K158	K63 Ub	ubiquitination	TRAF2, TRAF6, TRIM8	+	TNF-α, IL-1β, TGF-β	[52,53,54,55]
				?	+	Doxorubicin	[50]
				TRAF6, Ubc13	+	*H. pylori* infection (CagA)	[56]
			deubiquitination	USP4	–	Doxorubicin	[50]
	T178	P	autophoshporylation		+	IL-1β, TAB1 co-expression, etc.	[57]
	T184	P	autophosphorylation		+	TAB1 co-expression, etc.	[19]
		Ac	Acetylation	YopJ	–	bacterial infection	[58]
	T187	P	autophosphorylation		+	TAB1 co-expression, etc.	[57]
			phosphorylation	TPL-2	+	IL-17	[59]
			dephosphorylation	DUSP14 (MKP6)	–	TNF-α, IL-1β	[60]
				PP6, PP2Cβ-1, PP2Cε	–	IL-1β	[61,62,63]
				PP2A, calcineurin	–	TGF-β	[64,65]
		Ac	Acetylation	YopJ	–	bacterial infection	[58]
	S192	P	autophosphorylation	?	+	IL-1 *, TAB1 co-expression, etc.	[66]
	K209	K63 Ub	ubiquitination	TRAF6, Ubc13	+	IL-1 *	[67]
				?	+	Sef-S expression	[68]
	S412	P	phosphorylation	PKA	+	TNF-α, RANKL	[69]
				PKACα, PRKX	+	IL-1β, LPS, TNF-α	[70]
			dephosphorylation	PP1	–	TLR ligands	[71]
	S427	*O*-GlcNAc	*O*-GlcNAcylation	OGT	+	IL-1α, osmotic stress	[72]
	K562	K63 Ub	ubiquitination	TRAF6	+	LPS	[73]
			deubiquitination	USP18	−	LPS, TNF-α, TCR ligands	[74,75]
			deubiquitination	USP4, CYLD	–	TNF-α	[76,77,78]
TAB1	K294/319/335/350	K63 Ub	ubiquitination	MEKK1 (PHD), UBE2N	+	TGF-β	[79]
	S395	*O*-GlcNAc	*O*-GlcNAcylation	OGT	+	IL-1α, osmotic stress	[80]
	S423/T431	P	phosphorylation	p38α	?	LPS, IL-1α, TNF-α, H_2_O_2_, UV-C, anisomycin, sorbitol	[5]
	S438	P	phosphorylation	ERK, JNK	?	LPS, IL-1α, IL-1β, TNF-α, H_2_O_2_, UV-C, anisomycin, sorbitol	[5,81]
			dephosphorylation	DUSP14 (MKP6)	–	TCR ligands	[82]
	S452/453/456/457	P	phosphorylation	TAK1, p38	?	IL-1α, anisomycin, sorbitol	[83]
			dephosphorylation	calcineurin	?	?	[65]
		K48 Ub	ubiquitination	ITCH	–	TNF-α	[84]
TAB2	K329	SUMO	SUMOylation	PIAS3	–	?	[85]
	S372/S524	P	phosphorylation	?	?	IL-1β	[63]
	T456	*O*-GlcNAc	*O*-GlcNAcylation	OGT	?	?	[80]
	C673	Me	Methylation	NleE	–	bacterial infection	[86,87]
		K48 Ub	ubiquitination	RBCK1	–	IL-1β, TNF-α	[78]
TAB3	S60/T404	P	phosphorylation	p38α	?	IL-1α, IL-1β	[81]
	S408	*O*-GlcNAc	*O*-GlcNAcylation	OGT	+	IL-1β	[88]
	S506	P	phosphorylation	p38α, MAPKAP-K2/K3	?	IL-1α, IL-1β	[81]
	C692	Me	Methylation	NleE, OspZ	–	bacterial infection	[89,90]
		K48 Ub	ubiquitination	RBCK1	–	IL-1β, TNF-α	[91]

*: The subtype of IL-1 was not defined.
